# The effect of standard dose multivitamin supplementation on disease progression in HIV-infected adults initiating HAART: a randomized double blind placebo-controlled trial in Uganda

**DOI:** 10.1186/s12879-015-1082-x

**Published:** 2015-08-19

**Authors:** David Guwatudde, Molin Wang, Amara E. Ezeamama, Danstan Bagenda, Rachel Kyeyune, Henry Wamani, Yukari C. Manabe, Wafaie W. Fawzi

**Affiliations:** School of Public Health, Makerere University College of Health Sciences, Kampala, Uganda; Departments of Epidemiology and Biostatistics, Harvard School of Public Health, Channing Division of Network Medicine, Harvard Medical School, Boston, MA USA; Department of Epidemiology and Biostatistics, University of Georgia, Athens, GA USA; Department of Global Health and Population, Harvard School of Public Health, Boston, MA USA; Infectious Diseases Institute, Makerere University College of Health Sciences, Kampala, Uganda; Division of Infectious Diseases, Department of Medicine, John Hopkins University, Baltimore, MD USA; Departments of Global Health and Population, Nutrition and Epidemiology, Harvard School of Public Health, Boston, MA USA

## Abstract

**Background:**

Efficacy trials investigating the effect of multivitamin (MV) supplementations among patients on Highly Active Antiretroviral Therapy (HAART) have so far been inconclusive. We conducted a randomized, double blind, placebo controlled trial to determine the effect of one recommended daily allowance (RDA) of MV supplementation on disease progression in patients initiating HAART.

**Methods:**

Eligible subjects were randomized to receive placebo or MV supplementation including vitamins B-complex, C and E. Participants were followed for up to 18 months. Primary endpoints were: change in CD4 cell count, weight and quality of life (QoL). Secondary endpoints were: i) development of a new or recurrent HIV disease progression event, including all-cause mortality; ii) switching from first- to second-line antiretroviral therapy (ART); and iii) occurrence of an adverse event. Intent-to-treat analysis, using linear regression mixed effects models were used to compare changes over time in the primary endpoints between the study arms. Kaplan-Meier time-to-event analysis and the log-rank test was used to compare HIV disease progression events and all-cause mortality.

**Results:**

Four hundred participants were randomized, 200 onto MV and 200 onto placebo. By month 18, the average change in CD4 cell count in the MV arm was 141 cells/uL compared to 147 cells/uL in the placebo arm, a mean difference of −6 · 17 [95 % CI −29 · 3, 16 · 9]. The average change in weight in the MV arm was 3 · 9 kg compared to 3 · 3 kg in the placebo arm, a mean difference of 0 · 54 [95 % CI −0 · 40, 1 · 48]; whereas average change in QoL scores in the MV arm was 6 · 8 compared to 8 · 8 in the placebo arm, a mean difference of −2.16 [95 % CI −4 · 59,0 · 27]. No significant differences were observed in these primary endpoints, or in occurrence of adverse events between the trial arms.

**Conclusions:**

One RDA of MV supplementation was safe but did not have an effect on indicators of disease progression among HIV infected adults initiating HAART.

**Trial registration:**

Clinical trials NCT01228578, registered on 15th October 2010.

## Background

Vitamins and minerals are important for maintaining an optimal and responsive immune system [1, 2]. Micronutrient deficiencies and their effects on immune function and disease progression have been documented in HIV-infected persons [3]. These deficiencies are prevalent even before the development of symptoms of disease and are associated with accelerated HIV disease progression [4–6]. A number of studies conducted among HIV-infected adults during the pre-highly active antiretroviral therapy (HAART) era, have shown that multivitamin supplementation enhances immune reconstitution, reduces viral load, improves overall clinical outcomes, and reduces mortality [7–14]. However, only a few trials have explored the effect of multivitamin supplementation in relation to HIV progression during HAART, and some have reported beneficial treatment outcomes [15–17].

A recent trial conducted in Tanzania among HIV-infected adults initiating HAART compared the effect of multiple recommended daily allowance (RDA) doses of multivitamin supplements including vitamins B-complex, C and E, to a single RDA. Although the trial was stopped early because of negative side effects, at the time of stopping the trial at a median follow-up time of 15 months, multiple RDA supplementation was no different from a single RDA on several key measures that reveal HIV disease progression and risk of death [18]. Because this trial did not use a placebo arm, it is plausible that individuals in both treatment arms had similar benefits or supplementation had no effect in either arm. Thus, in spite of widespread use of nutritional supplements among HIV-infected patients, it is unclear if the benefits of micronutrient supplementation observed in relatively healthy patients not yet on HAART would also be notable among HIV-infected patients on HAART. We conducted a randomized, double-blind, placebo-controlled trial comparing one RDA of multivitamin supplements to placebo to evaluate the benefit of multivitamin supplements on disease progression among HIV-infected patients on HAART in Uganda. Participants were provided with daily dose of one RDA of oral multivitamin supplements including vitamins B-complex, C and E, or placebo for 18 months, and the efficacy and safety of the supplements were examined.

## Methods

A detailed description of methods used in this trial has been reported elsewhere [19]. Here we only describe methods relevant for results presented in this article.

### Setting

The trial was conducted at the Infectious Diseases Institute (IDI) in Kampala, Uganda. IDI provides out-patient care and treatment for HIV-infected persons. Over 10,000 people receive care through IDI, approximately 6000 of which are on HAART.

### Eligibility

Patients were recruited between April 2010 and June 2012, and eligible subjects had to meet the following inclusion criteria: a) at least 18 years old, b) HIV positive, c) initiating HAART at the time of randomization or had been on HAART for no longer than 6 months, d) had no intention of re-locating more than 20 km outside of the IDI within the next 18 months, e) agreed to allow home visits, and follow-up contacts as part of the trial, and f) provided written informed consent. Subjects with any of the following were excluded: a) women with a positive pregnancy test (who are provided iron and folic acid supplementation as prenatal standard of care per the Uganda Ministry of Health standards); b) individuals who were too ill to provide consent.

### Randomization and masking

Prior to initiation of the trial, a staff not associated with implementation of the trial at Harvard School of Public Health (HSPH), generated serial numbers from 1 to 400 and randomly assigned to intervention or placebo groups, in blocks of 10 participants. Trial regimen bottles (manufactured on special order from Tishcon Corp, Salisbury, MD), were then labeled with the appropriate participant serial numbers only. The regimen bottles were shipped to Uganda, but the list showing the trial arm to which each serial number was assigned remained anonymous at HSPH and was not accessible to trial staff in Uganda until the trial was un-blinded. The trial pharmacist dispensed the assigned regimen bottles to the participants in sequential order of enrollment. The intervention arm consisted of a daily dose of one RDA of oral multivitamins at a single RDA (based on the United States of America’s Food and Nutrition Board, Institute of Medicine, National Academies) [20, 21], of the following micronutrient formulations: B-complex vitamins (1 · 4 mg B1, 1 · 4 mg B2, 1 · 9 mg B6, 2 · 6 mcg B12, 18 mg niacin, 0 · 4 mg folic acid), 70 mg of vitamin C, and 10 mg of vitamin E; whereas the placebo consisted of an inactive pill of the same size, packaging, and coloration as the active multivitamin tablets. Participants were followed for up to 18 months with evaluations at baseline, months 3, 6, 12, and 18. Except the trial statistician who had to conduct interim analyses to facilitate the Data Safety Monitoring Board reviews; trial participants, trial staff, and the investigators were all blinded to the treatment allocation until the trial was un-blinded in March 2014.

### Measurements

Baseline social, demographic, and clinical characteristics of participants were recorded. During follow-up and at each study visit, medical officers performed a complete clinical examination, recorded all diagnostic tests performed and any medications prescribed. They also documented whether participants had attended additional clinic visits outside their scheduled study appointments, recording reasons, diagnoses, and any medications received outside those prescribed in the trial, as well as any hospitalizations and/or any heakltbh related adverse events that might have occurred.

#### Laboratory investigations

A blood specimen was taken from each participant at enrolment and every 6 months thereafter to measure CD4 cell counts, complete blood count, bilirubin, creatinine, and alanine transaminase (ALT) as part of clinical standard of care. Outside of standard of care measurements, we repeated CD4 cell count measurement at month 3 to enable us investigate any acute changes in CD4 cell count following randomization.

#### Quality of life

Quality of life (QoL) of participants was assessed at baseline and every 6 months thereafter, using the validated, culturally-adapted local language version of the Medical Outcomes Survey-HIV (MOS-HIV) [22]. This QoL survey consisted of the following ten subscales: perceived health (five items), pain (two items), QoL (one item), health transition (one item), role functioning (two items), social functioning (one item), physical functioning (six items), cognitive functioning (four items), health distress (three items), energy (four items). Item scores were arranged such that for each item, a higher score corresponded to better QoL. The QoL score of a subscale was computed as the sum of all the item scores under the subscale.

There is no published information about how to compute an overall QoL score for the MOS-HIV. We therefore considered the following two ways for computing the overall QoL score: 1) performing a linear transformation (i.e. subscale score multiplied by ten) so that the perfect score of each subscale is 10, then summing up the transformed subscale scores, 2) summing up the ten subscale scores to get an overall score, then performing the linear transformation on the overall score so that the perfect overall score is 100. We then compared the Coefficient of Variation (CV) of the overall QoL scores at baseline based on the two definitions above, and found that the first one was associated with a smaller CV. We therefore used the first definition for the overall QoL score.

##### Adherence

At each monthly drug refill visit, research nurses assessed compliance to the treatment (ART and trial regimens) through two ways, by direct questioning and recording patient self reported compliance to the medications, and using a pill count.

#### Trial endpoints

The primary endpoints were changes over time in absolute CD4+T cell count, weight, and QoL. Secondary endpoints were: i) development of a new or recurrent HIV disease progression event, including all cause mortality; ii) switching from 1st line to 2nd line HAART regimen; and iii) occurrence of an adverse event, including peripheral neuropathy, severe anemia, and diarrhea.

### Ethical compliance

The trial protocol was approved by the Scientific Review Committee of IDI; and the Institutional Review Boards of HSPH, and that of Makerere University School of Public Health. A Data Safety Monitoring Board was setup at the beginning of the trial, and monitored the trial every 6 months within the first year of initiation of the trial, and annually subsequently.

### Statistical analysis

#### Baseline comparisons

We compared the distribution of participants at enrollment in the two trial arms along baseline socio-demographic, and clinical characteristics using the chi-square or Fisher’s exact test for categorical variables, and the *t*-test or Wilcoxon rank sum test for continuous variables.

#### Primary endpoints analysis

Analyses for primary endpoints were based on the intention-to-treat analysis strategy. We applied linear mixed effects modeling with a random intercept, unstructured correlation matrix and robust standard errors, to evaluate changes over time in the primary outcomes in relation to the assigned treatment group. Visit month (categorical) at which the outcome was measured, treatment group and their interactions were included in the model as fixed effects. *P*-values for change over time were obtained through the contrast statements in SAS PROC MIXED, testing the null hypothesis that the mean changes at all the post-baseline visit months were equal between the treatment groups. The alternative hypothesis was that the mean changes were not equal at, at least one post-baseline visit month.

#### Secondary endpoints analysis

We examined a number of secondary endpoints that are indicative of HIV disease progression:

- Occurrence of specific HIV disease progression events and all cause mortality between the trial arms using Kaplan-Meier time-to-event analysis, the log-rank test, and Cox’s proportional hazard modeling. HIV progression events monitored included: *Pneumocystis carinii* pneumonia, cryptococcal meningitis, cytomegaloviral retinitis, mucocutaneous herpes simplex virus, histoplasmosis, esophageal candidiasis, extrapulmonary tuberculosis, lymphoma, Kaposi sarcoma, pulmonary *M. tuberculosis*, and recurrent pneumonia.
- Percentage of switching from first line, to second line antiretroviral (ARV) regimen, between the trial arms using the Fisher’s exact test.
- Occurrence of adverse events between the trial arms using Fisher’s Exact test, and mixed effects modeling with a logit link. The adverse events monitored included severe anemia (HB ≤7 g/dL), nausea, vomiting, high ALT levels (≥200 IU/I), peripheral neuropathy and diarrhea.

We also compared occurrence of post-randomization hospitalizations between the trial arms using the generalized estimating equation method, with log-link, an exchangeable correlation structure, and robust variance estimates; and the change over time in hemoglobin (HB), and ALT levels between the trial arms using linear mixed effects model analysis.

Finally, given some imbalance in the participants’ age between the trial arms at baseline, age was adjusted for in all model-based analyses. In the linear mixed effects model analyses for endpoints that constituted changes from baseline, the baseline value of the outcome was also adjusted for. For primary endpoints, we checked for effect modification by baseline values of CD4 cell count, BMI, and baseline ART use status (HAART naïve, on treatment for less or equal to 3 months, or on treatment for 3 to 6 months). We also examined effect modification by post-randomization multivitamin use prescribed outside of the trial as a time varying variable (1 = once taken, from then onwards assumed to be on multivitamins outside of the trial; and =0 for periods before taking). All tests were two-sided, statistical significance level was set to 0 · 05, and all analyses were performed using SAS version 9 · 0.

## Results

### Enrollment and follow-up

**Fig. 1:**
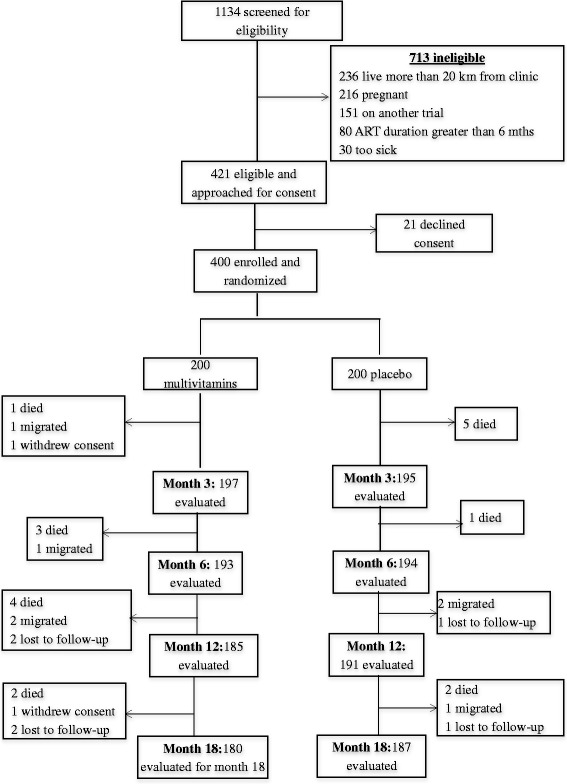
Trial schema

Between April 2010 and June 2011, we enrolled a total of 400 participants in this trial. Overall, 18 participants died during follow-up (ten in the multivitamin, and eight in the placebo arm), seven migrated to outside the study area (four in the multivitamin, and three in the placebo arm), six were lost to follow-up (four in the multivitamin, and two in the placebo arm), and one participant in the multivitamin arm voluntarily withdrew from the trial. The minimum follow up time was 1 · 5 months, a maximum of 18 months, and a median of 18 months. No significant differences were observed in the median follow-up times between the trial arms (*p* = 0 · 432). Figure 1 summarizes the trial schema.

### Baseline characteristics of trial participants

The majority of the participants, 227 (69 %) were female, 290 (72 %) were older than 30 years with an overall average of 35 · 8 years, and standard deviation (SD) of 9 · 0. Only 88 participants (22 %) reported using multivitamins prior to enrollment into the trial for a median duration of 1 month, and 200 (50 %) had been initiated on HAART within the past 6 months for a median duration of 1 · 9 months (Interquartile range = 0 · 5–3 · 9). Except for age, the trial arms were comparable at baseline with respect to socio-demographic characteristics, duration of ART use prior to randomization, and duration of multivitamin use prior to randomization (Table 1). The average age of the participants in the multivitamin arm was 36 · 9 years (SD = 9 · 6), compared to 34 · 7 years (SD = 8 · 1) in the placebo arm.

**Table 1:** Baseline characteristics of trial participants by treatment arm^a^

Characteristic		MV^a^ (*n* = 200)	Placebo (*n* = 200)
Sex	Males	57 (28.5 %)	66 (33.0 %)
	Females	143 (71.5 %)	134 (67.0 %)
Age (years)	18–20	9 (4.5 %)	1 (0.5 %)
	21–30	42 (21.0 %)	58 (29.0 %)
	31–40	85 (42.5 %)	100 (50.0 %)
	>40	64 (32.0 %)	41 (20.5 %)
	Mean ± SD^b^	36.9 ± 9.6	34.7 ± 8.1
Level of education	None	7 (3.0 %)	6 (3.0 %)
	Primary	103 (51.5 %)	103 (51.5 %)
	Secondary	70 (35.0 %)	71 (35.5 %)
	> Secondary	20 (10.0 %)	20 (10.0 %)
Marital status	Never Married	19 (9.5 %)	22 (11.0 %)
	Married/cohabiting	86 (43.0 %)	83 (41.5 %)
	Separated/divorced	64 (32.0 %)	59 (29.5 %)
	Widowed	31 (15.5 %)	36 (18.0 %)
HB (g/dL)	≤11.0	45 (22.5 %)	46 (23.0 %)
	>11.0	155 (77.5 %)	154 (77.0 %)
	Median (IQR)^b^	12.2 (11.2–13.2)	12.3 (11.3–13.5)
BMI (kg/m-sq)	<18.5	15 (7.5 %)	16 (8.0 %)
	18.5–24.9	122 (61.0 %)	136 (68.0 %)
	25.0–29.9	43 (21.5 %)	29 (14.5 %)
	≥30	17 (8.5 %)	19 (9.5 %)
	Missing	3 (1.5 %)	0 (0.0 %)
Time on ART (months)	0	93 (46.5 %)	108 (54.0 %)
	1–2	60 (30.0 %)	57 (28.5 %)
	3–6	47 (23.5 %)	35 (17.5 %)
	Median (IQR)^b^	1 (0–2)	0 (0–1)
CD4 count (cells/mm^3^)	≤50	24 (12.0 %)	39 (19.5 %)
	51–100	42 (21.0 %)	40 (20.0 %)
	101–200	77 (38.5 %)	73 (36.5 %)
	>200	53 (26.5 %)	46 (23.0 %)
	Missing	4 (2.0 %)	2 (1.0 %)
	Median (IQR)^b^	144.5 (86.0–215.3)	137.0 (68.0–192.0)
MV use duration (months)	0	159 (79.5 %)	153 (76.5 %)
	1–2	36 (18.0 %)	42 (21.0 %)
	>2	5 (2.5 %)	5 (2.5 %)

^a^ *ART* antiretroviral therapy, *HB* Hemoglobin, *MV* Multivitamin supplementation

^b^Variable treated as continuous variable

### Effect of multivitamin supplementation on primary endpoints

**Fig. 2:**
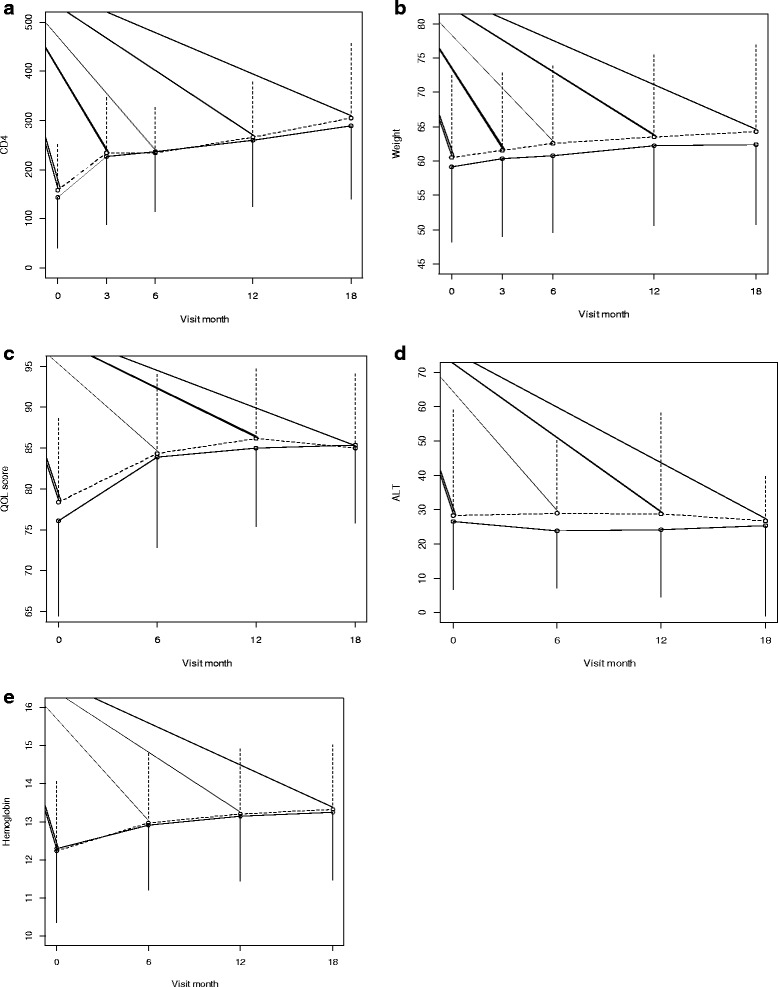
Trends in mean values of: CD4 cell count, weight, QoL scores, ALT and HB by trial arm. *Solid line* Multivitamin arm, *broken line* Placebo

No significant differences were observed in changes of the primary endpoints between the trial arms. In the multivitamin arm, the observed average increase in CD4 cell count between baseline and month 18 was 141 cells/uL, compared to 147 cells/uL in the placebo arm (*p*-value for change over time =0 · 726). Regarding weight, the observed average change in weight between baseline and month 18 was 3 · 9 kg in the multivitamin arm, compared to 3 · 3 kg in the placebo arm (*p*-value for change over time = 0 · 691). The observed average change in QoL scores between baseline and month 18 was 6 · 8 in the multivitamin arm, compared to 8 · 8 in the placebo arm (*p*-value for change over time = 0 · 454) (Table 2 and Fig. 2). Effect modification by post-randomization multivitamin use prescribed outside of the trial on the effect of the intervention on any of the three primary endpoints was not statistically significant. The effect of multivitamins was also not modified by baseline CD4 cell count, weight, age, or HAART status at baseline.

**Table 2:** Observed mean changes by visit month and treatment group for primary endpoints, ALT and HB^a^

End point	Multivitamin		Placebo		Two-sided *p*-value for change over time^b^
	- *n* -	Mean (SD)	- *n* -	Mean (SD)	
Change in CD4 (cells/uL)					
0–3 months	194	77 (97)	193	86 (103)	0.726
0–6 months	191	74 (95)	190	94 (92)	
0–12 months	183	106 (112)	188	117 (116)	
0–18 months	181	141 (160)	186	147 (130)	
Change in weight (kg)					
0–3 months	194	1.2 (2.9)	195	1.2 (3.3)	0.691
0–6 months	191	2.1 (4.2)	190	1.7 (4.0)	
0–12 months	183	3.2 (5.4)	188	3.1 (5.0)	
0–18 months	181	3.9 (6.2)	187	3.3 (5.8)	
Change in QoL score					
0–6 months	191	6.1 (11.1)	190	7.5 (12.7)	0.454
0–12 months	181	8.1 (10.9)	187	8.4 (12.0)	
0–18 months	181	6.8 (12.4)	186	8.8 (12.4)	
Change in ALT (IU/I)					
0–6 months	190	0.99 (32.8)	188	−2.78 (20.9)	
0–12 months	183	0.56 (41.5)	187	−1.66 (22.1)	0.064
0–18 months	180	−1.69 (33.4)	186	−0.02 (30.0)	
Change in HB (g/dL)					
0–6 months	191	0.68 (1.6)	190	0.60 (1.5)	
0–12 months	183	0.92 (1.4)	188	0.81 (1.6)	0.977
0–18 months	181	1.01 (1.5)	186	0.91 (1.7)	

^a^ *ALT* Alanine transaminase, *HB* Hemoglobin, *Q1* Upper limit of first quartile, *Q3* Upper limit of third quartile

^b^Based on linear mixed effects model analysis adjusting for age and baseline value

### Effect of multivitamin supplementation on secondary endpoints

The observed average change in hemoglobin levels between baseline and month 18 was 1 · 01 g/dL in the multivitamin arm, compared to 0 · 91 g/dL in the placebo arm (*p*-value for change over time = 0 · 977). Similarly, the average change in ALT levels between baseline and month 18 was −1 · 69 IU/I in the multivitamin arm, compared to −0 · 020 IU/I in the placebo arm (*p*-value for change over time = 0 · 064).

**Fig. 3:**
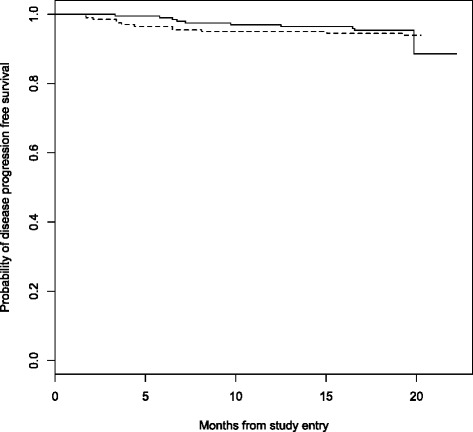
Kaplan-Meier plots for confirmed HIV disease progression events or all-cause death. *Solid line* Multivitamin arm, *broken line* Placebo

The confirmed post randomization HIV disease progression events observed in this trial were: five cases of extra pulmonary tuberculosis (all in the placebo arm), and one case of Kaposi sarcoma in the intervention arm. Figure 3 shows the Kaplan-Meier plot of the time to a confirmed post-randomization HIV disease progression event or death (whichever occurred first), by trial arm. There was no significant difference in occurrence HIV disease progression events and all cause mortality between the trial arms (*p*-value = 0 · 615, HR = 0 · 81, 95 % CI = [0 · 53–2 · 88]).

A total of 35 post-randomization hospitalization events were observed among 34 participants in the multivitamin arm, compared to 39 among 35 participants in the placebo arm. There was no difference in occurrence of these events between the trial arms (Risk Ratio = 0 · 94, 95 % CI = 0 · 61–1 · 47). Only 2 participants in the placebo arm were switched from a first line to a second line anti-retroviral regimen (Fisher’s Exact test *p*-value = 0.479).

A total of 550 adverse events were observed in this trial that included one event of severe anemia in the multivitamin arm, and one in the placebo arm (*p* = 0 · 995), 345 events of nausea (153 in the multivitamin and 192 in the placebo arm; *p* = 0 · 088), 200 cases of vomiting (84 in the multivitamin, and 116 in the placebo arm; *p* = 0 · 058), and one event of high ALT (>200 IU/I) in the multivitamin arm compared to none in the placebo arm; *p* = 0 · 496, (Table 3).

**Table 3:** Adverse events in the multivitamin and placebo arms^a^

		Multivitamin		Placebo		*P*-value
Endpoint	Month	*n*	# of events (%)	*n*	# of events (%)	
Severe anemia (HB <7 g/dL)	6	191	1 (0.5 %)	190	0	0.995^b^
	12	183	0	188	0	
	18	181	0	187	1 (0.5 %)	
Nausea	1–3	194	62 (32.0 %)	195	79 (40.5 %)	0.088^c^
	4–6	191	38 (19.9 %)	190	44 (23.2 %)	
	10–12	183	25 (13.7 %)	188	33 (17.6 %)	
	16–18	181	28 (15.5 %)	187	36 (19.3 %)	
Vomiting	1–3	194	34 (17.5 %)	195	52 (26.7 %)	0.058^c^
	4–6	191	18 (9.4 %)	190	23 (12.1 %)	
	10–12	183	15 (8.2 %)	188	22 (11.7 %)	
	16–18	181	17 (9.4 %)	187	19 (10.2 %)	
High ALT (>200 IU/I)	6	191	0	190	0	0.496^b^
	12	183	1 (0.5 %)	188	1 (0.5 %)	
	18	181	0	187	1 (0.5 %)	

^a^ *ALT* Alanine transaminase, *HB* Hemoglobin

^b^From Fisher’s exact test for 2 × 2 table

^c^Mixed effects model analysis using logit link

## Discussion

We examined the effect of one RDA dose of multivitamin supplementation including vitamins B-complex, C, and E on disease progression among HIV-infected adults in Uganda initiating HAART and observed no overall effect on the primary endpoints, nor in the secondary endpoints.

Previously, in several placebo-controlled trials and high quality large observational studies, multivitamins provided in single RDA or multiples of RDA resulted in slower disease progression and mortality among HIV infected individuals who were in earlier stages of disease and had not yet been initiated on HAART. These studies have been carried out in the U.S., Thailand, Tanzania, and South Africa, among other settings [23]. In a recently published randomized controlled trial from Botswana [10], these findings were confirmed among pre-HAART patients among whom micronutrient supplementation that included multivitamins and selenium for 24 months was found to be safe and significantly delayed disease progression.

We wanted to examine the efficacy of nutritional supplements as adjunct to HAART, given the high mortality and risk of opportunistic infections in the first several months of antiretroviral therapy particularly in those with low CD4 T cell counts [24]. A previous trial conducted by our team in Tanzania to investigate the effect of a similar combination of multivitamin supplements (B-complex, C and E) found no difference in the effect of a multiple dose versus one RDA multivitamin supplementation on HIV disease progression or death among HIV-infected adults initiating HAART [18]. Of concern in that trial, high dose multivitamins supplementation resulted in a significant elevation of liver enzyme ALT, and there was a suggestion of higher mortality among individuals who had BMI <16 kg/m^2^ [18]. In the current trial from Uganda, the single RDA dose of multivitamins was safe, with no difference compared with placebo in risk of elevated liver enzymes and other safety endpoints.

While micronutrients supplements are beneficial among individuals in stages prior to the initiation of HAART [10, 23], it may be that these nutrients are not sufficient and the addition of macronutrients is critical with advanced disease stage. In a recently published trial from Ethiopia, lipid based supplements containing essential fatty acids, protein, in addition to multivitamins provided for 3 months at the initiation of HAART resulted in a significant improvement in lean body mass and total weight gain compared with individuals who did not receive the nutritional supplement [25].

We examined whether timing of provision of nutritional supplements relative to HAART initiation provides differential effects. Based on work in Zambia, Koethe and Heimburger proposed that with increased appetite and nutritional intake after HAART initiation, a precipitous decline in serum phosphate may occur contributing to higher early mortality [26]. In the trial from Ethiopia noted above, individuals provided with nutritional supplement in the second 3 months after initiation of HAART had significant better weight gain compared with those who received the intervention in the first 3 months [25], suggesting that delay in provision of nutritional intervention may result in better outcomes. In our Uganda trial, we found no difference in the effect of multivitamins on any of the primary outcomes whether patients where HAART naïve or had been on therapy for up to 6 months.

The trial has several strengths and limitations. This is the first placebo-controlled trial to examine the efficacy of multivitamin supplements among individuals who are on antiretroviral therapy in an African setting. The trial from Tanzania compared one RDA with multiples of the RDA of the same vitamins (B-complex, C and E) [18], and thus it was not possible to exclude the possibility that both single and multiple RDA dose regimens had equivalent effects.

Participants in this trial from Uganda were relatively well-nourished group at baseline as evidenced by the fact that only 7 · 8 % were under-weight (BMI <18 · 5 kg/m^2^). It is possible that there is minimal benefit to nutritional supplementation among participants with controlled disease. Thus, the findings of the trial may not be generalizable to populations where severe malnutrition is more prevalent with advanced HIV disease.

We note that our trial with a sample size of 400 was relatively small and was powered to examine the effect of multivitamin supplementation on only three endpoints. It is possible that the trial did not have enough power to detect differences in the effect of the multivitamin supplementation on less common endpoints (such as incidence of severe immunosuppression or wasting) that may indicate HIV advanced disease progression, and to examine effect modification by various participants’ characteristics. Larger trials may be necessary to confirm or dispel our findings.

## Conclusions

This trial found that single dose RDA of MV supplementation did not have an effect on indicators of disease progression among HIV infected adults initiating HAART. However multivitamin supplements at single RDA dose are safe and can be provided to individuals on anti-retroviral therapy. Although our trial participants were relatively well nourished, malnutrition is endemic in many low income countries and is a common manifestation among HIV-infected individuals [27–29]. Micronutrient intakes at daily recommended levels need to be assured, either through diet or when needed through use of supplements [30]. Additional research is needed on the role of more comprehensive supplement including other essential macronutrients, as the benefits of these may be higher.
